# Recruitment of VPS33A to HOPS by VPS16 Is Required for Lysosome Fusion with Endosomes and Autophagosomes

**DOI:** 10.1111/tra.12283

**Published:** 2015-04-30

**Authors:** Lena Wartosch, Ufuk Günesdogan, Stephen C. Graham, J. Paul Luzio

**Affiliations:** ^1^ Cambridge Institute for Medical Research and Department of Clinical Biochemistry, Wellcome Trust/MRC BuildingUniversity of CambridgeCambridgeCB2 0XYUK; ^2^ Wellcome Trust/Cancer Research UK Gurdon InstituteUniversity of CambridgeCambridgeCB2 1QNUK; ^3^ Department of PathologyUniversity of CambridgeCambridgeCB2 1QPUK

**Keywords:** autophagy, CORVET, endocytosis, HOPS, lysosomes, SM protein, tethering factor, VPS16, VPS33A

## Abstract

The mammalian homotypic fusion and vacuole protein sorting (HOPS) complex is comprised of six subunits: VPS11, VPS16, VPS18, VPS39, VPS41 and the Sec1/Munc18 (SM) family member VPS33A. Human HOPS has been predicted to be a tethering complex required for fusion of intracellular compartments with lysosomes, but it remains unclear whether all HOPS subunits are required. We showed that the whole HOPS complex is required for fusion of endosomes with lysosomes by monitoring the delivery of endocytosed fluorescent dextran to lysosomes in cells depleted of individual HOPS proteins. We used the crystal structure of the VPS16/VPS33A complex to design VPS16 and VPS33A mutants that no longer bind each other and showed that, unlike the wild‐type proteins, these mutants no longer rescue lysosome fusion with endosomes or autophagosomes in cells depleted of the endogenous proteins. There was no effect of depleting either VIPAR or VPS33B, paralogs of VPS16 and VPS33A, on fusion of lysosomes with either endosomes or autophagosomes and immunoprecipitation showed that they form a complex distinct from HOPS. Our data demonstrate the necessity of recruiting the SM protein VPS33A to HOPS via its interaction with VPS16 and that HOPS proteins, but not VIPAR or VPS33B, are essential for fusion of endosomes or autophagosomes with lysosomes.

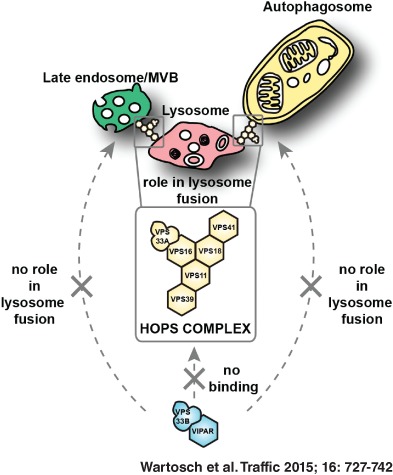

In mammalian cells, the transport of macromolecules to the lysosome depends on tightly regulated multi‐step processes involving bi‐directional vesicular transport and membrane fusion between distinct organelles. During endocytosis, mammalian cells take up cell‐surface membrane proteins, bound ligands, lipids and extracellular fluid into vesicles that subsequently fuse with early endosomes. These mature into late endosomes (also known as multivesicular bodies) that fuse with lysosomes to form endolysosomes 1, 2. Similarly, macroautophagy (hereafter referred to as autophagy) delivers intracellular macromolecules and organelles for degradation by lysosomal hydrolases via fusion of autophagosomes with lysosomes to form autolysosomes 3, 4. Much of the protein machinery required for traffic through the mammalian endocytic and autophagic pathways comprises homologs of proteins first identified in yeast on pathways to the vacuole, which is the yeast counterpart of the mammalian lysosome. In yeast, the heterohexameric homotypic fusion and vacuole protein sorting (HOPS) complex, comprising the vacuolar protein sorting (Vps) proteins Vps11p, Vps16p, Vps18p, Vps33p, Vps39p and Vps41p, has been shown to be a Rab GTPase‐dependent tethering complex required for fusion of vacuoles with transport vesicles or other vacuoles 5, 6, 7, 8, 9. Yeast HOPS aids in trans‐SNARE complex assembly, protects SNARE complexes from disassembly and promotes membrane fusion 10, 11, 12. In addition, yeast Vps39p has been reported to act independently of the remainder of the HOPS complex in creating contact sites between vacuoles and mitochondria 13, 14 and it is also proposed to play a role in controlling the activity of target of rapamycin complex 1 (TORC1) 15. Very little is known about how the metazoan HOPS complex functions at the mechanistic level, although functionally some or all HOPS proteins have been shown to be required for the maturation of endosomes 16, homotypic fusion of late endosomes 17, the delivery of endocytosed cargo to lysosomes 17, 18, 19, the biogenesis of lysosome related organelles [LROs; 18, 19, 20, 21], Ebola virus entry into cultured cells 22, the resistance of melanoma cells to cytotoxic agents 23, the fusion of phagosomes with lysosomes 24, 25, 26 and fusion of autophagosomes with lysosomes 27, 28, 29. There is also evidence that the mammalian HOPS protein VPS41 has additional functions that are independent of the complex in the fusion of lysosome‐associated membrane protein (LAMP) carriers with late endosomes 30, and the biogenesis of regulated secretory granules 31. In addition, it has been proposed that mammalian VPS18 may be involved in controlling the levels and localization of the serine/threonine‐specific protein kinase AKT2 on endosomes, thus potentially playing a role in the regulation of signalling pathways 32.

It is now known that a related complex, the class C core vacuole/endosome tethering (CORVET) complex, which is involved in tethering and fusion of early endosomes 33, 34, 35, 36, 37, 38, is present in *Saccharomyces cerevisiae*, *Caenorhabditis elegans* and mammals. HOPS and CORVET share four core subunits (Vps11p, Vps16p, Vps18p and Vps33p in yeast), with mammalian CORVET having the two accessory subunits VPS8 and TGFBRAP1/TRAP1 (homolog of yeast Vps3p) instead of VPS39 and VPS41 in HOPS 33, 37, 39. It is also apparent that metazoans have two paralogs of both yeast Vps33p and Vps16p, but there is currently a lack of clarity about whether both paralogs of VPS16 (VPS16/VPS16A and VIPAR/VIPAS39/SPE‐39/VPS16B) and VPS33 (A and B) can be part of HOPS and/or CORVET 33, 38, 40, 41.

VPS33A and VPS33B are members of the Sec1/Munc18 (SM) family of proteins that, together with SNAREs, comprise the core membrane fusion machinery in eukaryotes and interact with tethering complexes [reviewed in 42, 43]. Recently, we solved the crystal structure of the human SM protein VPS33A in complex with a fragment of VPS16 44, and a similar structure was solved by others with proteins from the thermophilic fungus *Chaetomium thermophilum* 45. We proposed that the structure of VPS33A in complex with VPS16 represents the archetypal interaction between SM proteins and tethering complexes 44. These studies have given us a structural framework to study the functional importance of the association between an SM protein and a tethering complex. We have therefore established cell‐based assays for both endosome‐ and autophagosome–lysosome fusion events to test the importance of the VPS33A/VPS16 interface, and thus recruitment of SM protein activity to the HOPS complex, for HOPS‐dependent membrane fusion. We have also addressed the question of whether VPS33B and VIPAR can functionally substitute for VPS33A and VPS16. Our results show that the association of human VPS33A with HOPS via its interaction with VPS16 is required for both endosome‐ and autophagosome–lysosome fusion. Further, we found that neither VPS33B nor VIPAR are required for these fusion events and are physically neither part of the human HOPS complex nor of the human CORVET complex.

## Results

### All components of the human HOPS complex are essential for fusion of endosomes and lysosomes but VIPAR and VPS33B are not required

**Figure 1:**
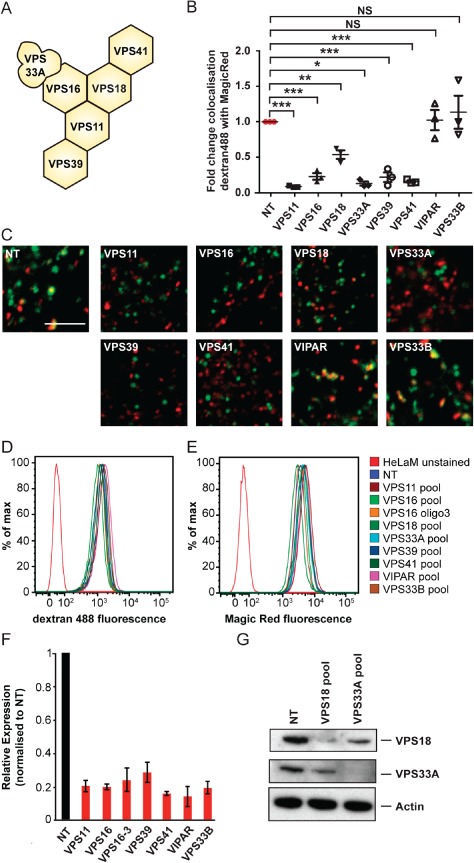
**siRNA depletion of HOPS components but not VIPAR or VPS33B inhibits the fusion of endosomes with lysosomes**. HeLaM cells were transfected twice with a single siRNA oligonucleotide or a pool of four siRNA oligonucleotides at 100 nm. A) Schematic model of the human HOPS complex 44. B) Colocalisation of dextran Alexa Fluor® 488 with Magic Red® using images captured by live‐cell confocal microscopy after knock‐down of HOPS proteins with siRNA oligonucleotide pools (NT: non‐targeting siRNA). Mean ± SEM of three independent experiments with five fields each, ≥30 cells total per condition. *p < 0.01, **p < 0.003, ***p < 0.0004, NS, not significant, using two‐tailed unpaired t‐test. C) Representative live‐cell confocal microscopy images of cells quantified in (B) that had been loaded with dextran Alexa Fluor 488 10,000 MW (green) for 2 h, chased for 1 h and then stained with Magic Red® (red) for lysosomes. Scale bar 5 µm. D) FACS analysis of dextran Alexa Fluor 488 fluorescence after uptake as in (B) and (C). E) FACS analysis of Magic Red® fluorescence of cells treated as in (B) and (C). For both (D) and (E), 30,000 cells were measured per condition, with representative traces shown for each condition from one of three independent experiments. Data were analysed using flowjo software and histogram overlays are displayed as %Max, scaling each curve to mode = 100%. F) RNA was purified from cells and transcribed into cDNA. The knock‐down efficiency was evaluated by quantitative real‐time PCR with gene‐specific primers (Table S1). Detection of Actin transcripts served as a reference. ΔΔCt values were calculated and relative transcript levels of three independent experiments are shown as mean ± SD. G) Protein lysates were analysed by immunoblotting with specific VPS18 or VPS33A antibodies to confirm knock‐down efficiencies. Actin served as loading control.

In order to study the function of human HOPS proteins in the regulation of membrane fusion in the late endocytic pathway of cultured cells we employed a modified version of a previously published protocol 17 to visualize the delivery of endocytosed fluorescent‐labelled dextran to enzymatically active endolysosomal/lysosomal compartments. In this assay, we depleted individual HOPS proteins in cultured HeLaM cells with small interfering RNA (siRNA) and quantified the mixing of endocytosed green fluorescent dextran Alexa Flour® 488, which had been chased into the endocytic pathway, with the red fluorescent cresyl violet product of hydrolysis of Magic Red®, a substrate for the lysosomal acid hydrolase Cathepsin B. Depletion of any of the components of human HOPS complex, VPS11, VPS16, VPS18, VPS33A, VPS39 or VPS41 (Figure 1A), inhibited fusion of dextran Alexa Flour 488‐positive endosomes with Magic Red®‐positive lysosomes (Figure 1B,C and Figure S1). In contrast, knock‐down of VIPAR or VPS33B, which previously were suggested to be alternative subunits for VPS16 and VPS33A in HOPS 40, 41, had no effect on the delivery of dextran Alexa Flour 488 from endosomes to lysosomes (Figure 1B,C). We determined by fluorescent activated cell sorting (FACS) analysis that the total fluorescence of both fluorescent markers was the same for all genotypes analysed (Figure 1D,E). These data are consistent with a previous study showing that depletion of VPS39 or VPS41 had no effect on fluorescent dextran uptake or the number of Magic Red®‐positive lysosomes despite impairing delivery of the dextran to lysosomes 17. The difference between the results that we obtained after depleting the HOPS proteins or VIPAR and VPS33B can neither be explained by different amounts of dextran Alexa Flour 488 uptake nor by differences in the overall Magic Red® signal from lysosomes. Analysis of the knock‐down efficiencies by quantitative real‐time PCR or immunoblotting indicated similar levels of depletion (∼80%) between all genotypes analysed (Figure 1F,G). Taken together, these data show that all human HOPS components, but not VIPAR or VPS33B, are required for the fusion of endosomes with lysosomes in HeLaM cells.

### Recruitment of VPS33A to the HOPS complex via interaction with VPS16 is required for endosome–lysosome fusion

**Figure 2:**
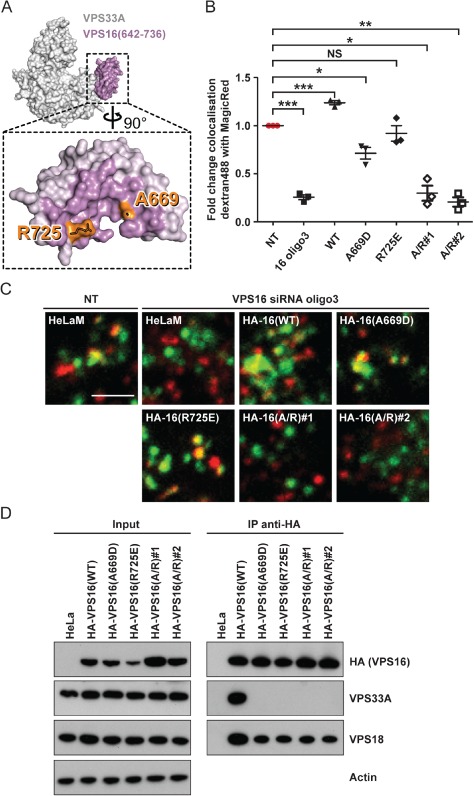
**The association of VPS33A with HOPS via VPS16 is essential for endosome–lysosome fusion**. Analysis of HeLaM cells or HeLaM cells stably expressing N‐terminally haemagglutinin (HA)‐tagged siRNA oligonucleotide 3 (oligo3)‐resistant VPS16 wild type (WT), single mutant (A669D, A725E), or double mutant A669D/R725E (A/R, two independent clonal lines, #1 and #2). A) Structure of VPS33A in complex with VPS16 residues 642–736 44. VPS33A and VPS16 are shown as white and purple molecular surfaces, respectively. Inset shows the binding footprint of VPS33A on VPS16 in darker purple, with residues essential for robust VPS33A binding highlighted in orange. Image was prepared using PyMOL. B and C) Cells were transfected with non‐targeting (NT) siRNA oligonucleotide or VPS16 siRNA oligo3 at 100 nm and subjected to live cell microscopy. Quantification of colocalisation of dextran Alexa Fluor® 488 (green) with Magic Red® (red) shown in (B). Mean ± SEM of three independent experiments with five fields each, ≥30 cells total per condition. *p < 0.01, **p < 0.003, ***p < 0.001, NS, not significant, using two‐tailed unpaired t‐test. Representative confocal microscopy images of cells quantified in (B) are shown in (C). Scale bar: 2.5 µm. D) Protein lysates were subjected to immunoprecipitation (IP) with anti‐HA affinity matrix and immunoblotting. Overexpressed HA‐VPS16 was detected with an anti‐HA antibody and endogenous VPS33A and VPS18 with specific antibodies. Actin served as loading control.

**Figure 3:**
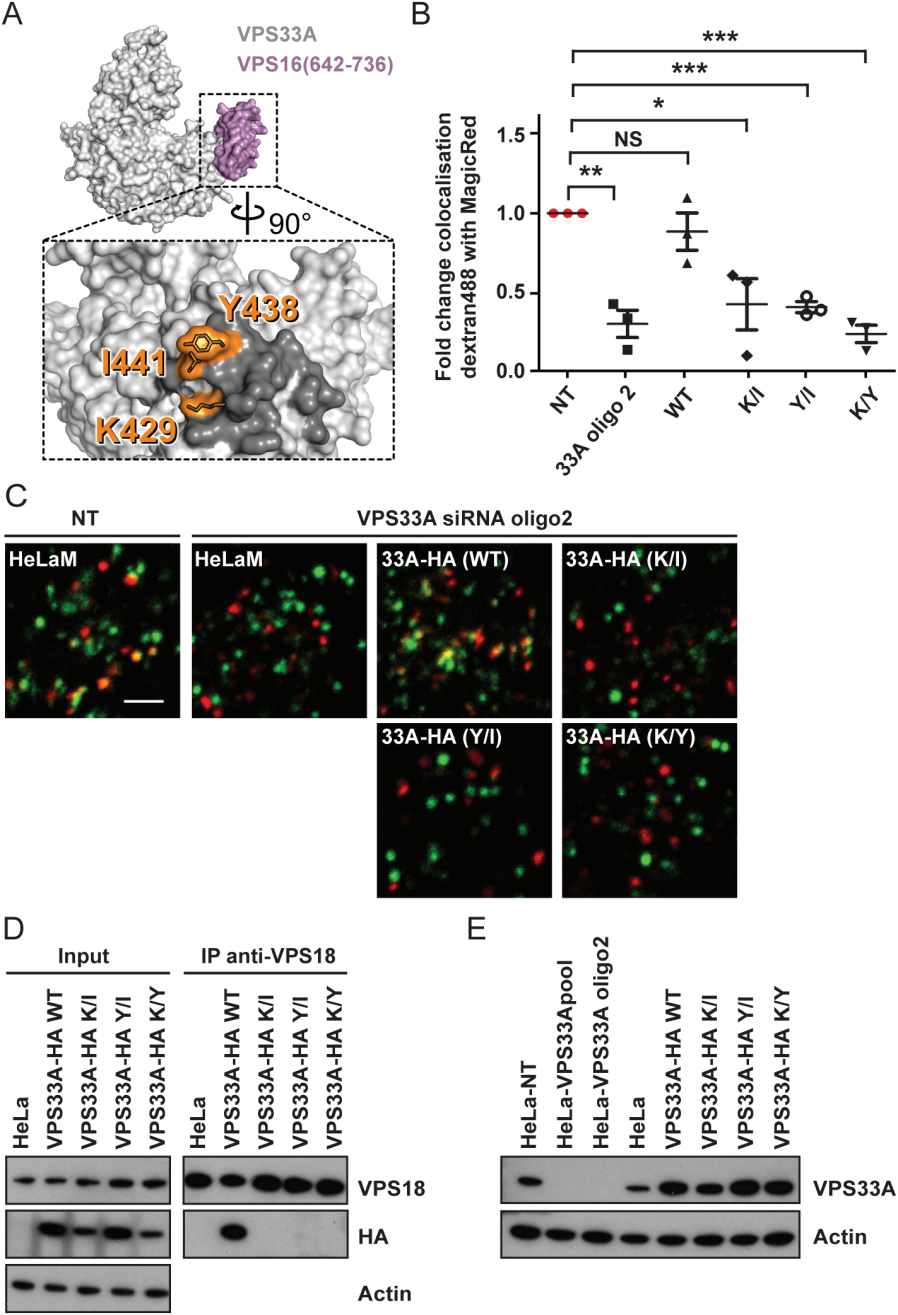
**VPS33A mutated at its binding interface with VPS16 cannot support endosome‐lysosome fusion**. Analysis of HeLaM cells or HeLaM cells stably expressing C‐terminally haemagglutinin (HA)‐tagged siRNA oligonucleotide 2 (oligo2)‐resistant VPS33A wild type (WT) or the double mutants K429D/I441K (K/I), Y438D/I441K (Y/I) or K429D/Y438D (K/Y). A) Structure of VPS33A in complex with VPS16 residues 642–736 44, coloured as in Figure 2A. Inset shows the binding footprint of VPS16 on VPS33A in darker grey, with residues essential for robust VPS16 binding highlighted in orange. Image was prepared using PyMOL. B and C) Cells were transfected with non‐targeting (NT) siRNA oligonucleotide or VPS33A siRNA oligo2 at 100 nm and subjected to live cell microscopy. Quantification of colocalisation of dextran Alexa Fluor® 488 (green) with Magic Red® (red) shown in (C). Mean ± SEM of three independent experiments with five fields each, ≥30 cells total per condition. *p < 0.02, **p < 0.002, ***p < 0.001, NS, not significant, using two‐tailed unpaired t‐test. Representative confocal microscopy images of cells quantified in (B) are shown in (C). Scale bar 2.5 µm. D) Protein lysates were subjected to immunoprecipitation (IP) with an anti‐VPS18 antibody and immunoblotting. Overexpressed HA‐VPS33A was detected with an anti‐HA antibody and endogenous VPS18 with a specific antibody. Actin served as loading control. E) Immunoblot of cell lysates from HeLaM cells, or from HeLaM cells stably overexpressing VPS33A‐HA(WT), VPS33A‐HA(K/I), VPS33A‐HA(Y/I) or VPS33A‐HA(K/Y), transfected twice with non‐targeting (NT) siRNA oligonucleotide as a control or a specific siRNA pool or single oligo2 to deplete endogenous VPS33A. VPS33A was detected with a specific VPS33A antibody. Actin served as a loading control. Expression levels, calculated as an increase compared to endogenous VPS33A in HeLaM cells, were: WT: ninefold, K/I: fourfold, Y/I: 14‐fold, K/Y: ninefold as measured by densitometric analysis of X‐ray films using imagej software.

To test whether the interaction of VPS33A and the rest of HOPS is required for endosome–lysosome fusion, we mutated in VPS16 the critical residues alanine 669 (A669D) and/or arginine 725 (R725E), which we have previously shown to be required for binding of VPS16 to VPS33A without substantially altering folding or stability [44, Figure 2A]. siRNA‐resistant HA‐tagged wild‐type HA‐VPS16(WT) or mutant HA‐VPS16(A669D), HA‐VPS16(R725E), or HA‐VPS16(A669D/R725E) were stably expressed in HeLaM cells. Expression of HA‐VPS16(WT) rescued the block in endosome–lysosome fusion observed upon depletion of endogenous VPS16 after transfection with a single siRNA oligonucleotide (oligo3; Figure 2B,C). Expression of VPS16 containing single mutations of either of the amino acids A669 or R725, present at the binding interface with VPS33A, also rescued the block in endosome–lysosome fusion, although the rescue was only partial when expressing the single mutant HA‐VPS16(A669D) (Figure 2B,C). This result may arise from residual binding of these VPS16 point mutants to VPS33A: we previously observed that purified VPS33A‐GST can pull‐down myc‐VPS16(A669D) and myc‐VPS16(R725E) produced by in vitro transcription/translation, although both are pulled down much less efficiently than is myc‐VPS16(WT) [see Fig. 5A in 44]. We conclude that our immunoprecipitation experiments (Figure 2D) are less sensitive than the previous pull‐down experiments in detecting the interaction of VPS16 with VPS33A, and that the presence of minute amounts of HOPS complex containing VPS33A, undetectable by immunoprecipitation, is sufficient to support delivery of the endocytosed dextran from endosomes to lysosomes. Expression of the double mutant HA‐VPS16(A669D/R725E), which also cannot immunoprecipitate endogenous VPS33A (Figure 2D), failed to rescue the block in endosome–lysosome fusion in two independent clonal HeLaM cell lines (Figure 2B,C). It should be noted that both of the single mutants of HA‐VPS16 and the double mutant were able to co‐immunoprecipitate VPS18 to a similar extent, consistent with them all being correctly folded and incorporated into the HOPS complex (Figure 2D). The block in endosome–lysosome fusion upon expression of the double mutant HA‐VPS16(A669D/R725E) in the cells depleted of endogenous VPS16 clearly shows that the association between VPS33A and VPS16 is required for endosome–lysosome fusion. This was confirmed when we mutated residues in VPS33A, lysine 429 (K429D), tyrosine 438 (Y438D) and isoleucine 441 (I441K), which we had previously shown to be required for the binding to VPS16 [44, Figure 3A]. siRNA‐resistant haemagglutinin (HA)‐tagged wild‐type VPS33A(WT)‐HA or three different double mutants, VPS33A(K429D/I441K)‐HA, VPS33A(Y438D/I441K)‐HA or VPS33A(K429D/ Y438D)‐HA, were stably expressed in HeLaM cells and their ability to support fusion of endosomes with lysosomes was analysed after depletion of endogenous VPS33A by transfection with a single oligonucleotide (oligo2; Figure 3B–C). As observed for VPS16, expression of VPS33A(WT)‐HA rescued the block in endosome–lysosome fusion detected upon depletion of endogenous VPS33A, whilst all three double mutants failed to rescue. Immunoblot analysis after immunoprecipitation with an anti‐VPS18 antibody and detection of the HA‐tagged proteins confirmed that the inability of VPS33A(K429D/I441K)‐HA, VPS33A(Y438D/I441K)‐HA and VPS33A(K429D/Y438D)‐HA to rescue the block in endosome–lysosome fusion was due to their loss of interaction with the remainder of the HOPS complex (Figure 3D). The inability of the double mutants to rescue when compared to VPS33A(WT)‐HA was not due to different levels of expression (Figure 3E). Taken together, our data demonstrate that the recruitment of VPS33A to the HOPS complex via its interaction with VPS16 is crucial for endosome–lysosome fusion in mammalian cells.

### VPS16 and VPS33A, but not VIPAR or VPS33B, are required for fusion of autophagosomes with lysosomes

**Figure 4:**
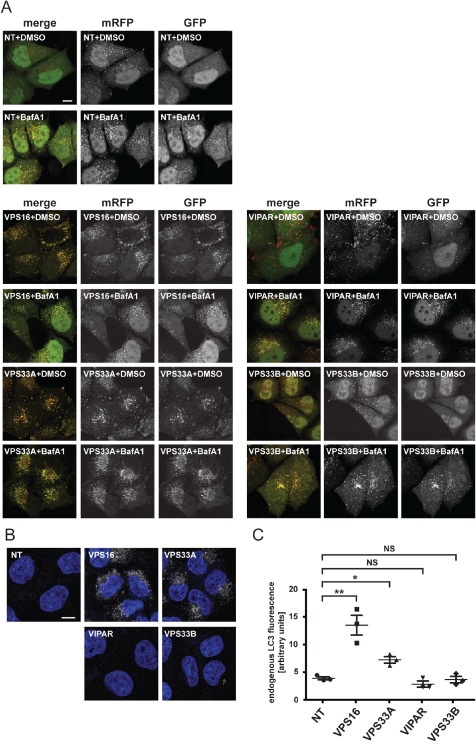
**siRNA depletion of the HOPS proteins VPS33A or VPS16 inhibits the fusion of autophagosomes with lysosomes**. Endogenous VPS16, VPS33A, VIPAR or VPS33B were depleted by transfection with specific siRNA oligonucleotides at 100 nm. Transfection with non‐targeting (NT) siRNA oligonucleotide served as control. A) Confocal images of HeLaM cells stably expressing the autophagy marker mRFP‐GFP‐LC3. Cells were treated with dimethyl sulphoxide (DMSO) (control) or 400 nm bafilomycinA1 (BafA1) to block autophagosome–lysosome fusion. Scale bar: 10 µm. B) Representative confocal images of fixed HeLa cells that were immunostained for endogenous LC3 (white). DNA (blue) was labelled with Hoechst 33258. Scale bar: 10 µm. C) For each condition, 10 fields (≥5 cells per field) were imaged for each of three independent experiments and the endogenous LC3 fluorescence signal per area measured using imagej. Mean ± SEM. *p < 0.04, **p < 0.01, NS, not significant calculated using two‐tailed unpaired t‐test.

All components of mammalian HOPS complex have previously been shown to be required in the final step of macroautophagy, the fusion of autophagosomes with lysosomes, although conflicting data has been reported for VPS16 28, 29, 46. Our depletion experiments described above led us to conclude that VIPAR and VPS33B do not play a role in endosome–lysosome fusion (Figure 1), but we also investigated whether they function in autophagosome–lysosome fusion (Figure 4). To this end, VPS16, VPS33A, VIPAR or VPS33B were depleted in HeLaM cells stably expressing the pH sensitive autophagy marker mRFP‐GFP‐LC3 47, 48. In these cells, because of the coincident fluorescent signal from both mRFP and GFP, autophagosomes appear yellow in merged confocal images. After the fusion of autophagosomes with lysosomes, the GFP fluorescence is quenched by acidic pH and autolysosomes show only a mRFP signal. The fusion of autophagosomes with lysosomes can be inhibited by a 6‐h incubation with 400 nm bafilomycinA1 49, which leads to the accumulation of yellow autophagosomes in contrast to control cells that show many red autolysosomes and only a small number of yellow autophagosomes (Figure 4A). Depletion of either VPS16 or VPS33A caused a significant accumulation of yellow autophagosomes in the HeLaM cells expressing mRFP‐GFP‐LC3 that was comparable to control cells transfected with non‐targeting siRNA and then treated with bafilomycinA1 (Figure 4A). In cells depleted of either VPS16 or VPS33A, treatment with bafilomycinA1 had no additional effect on the inhibition of autophagosome–lysosome fusion (Figure 4A). In contrast to depletion of VPS16 or VPS33A, knock‐down of VIPAR or VPS33B had no effect on autophagosome–lysosome fusion. These cells contained mainly red autolysosomes and only a few yellow autophagosomes, similar to non‐targeting siRNA treated cells (Figure 4A). Treatment of VIPAR or VPS33B knock‐down cells with bafilomycinA1 inhibited autophagosome–lysosome fusion, consistent with there being no disruption of the autophagy pathway upon depletion of VIPAR and VPS33B.

To characterize the autophagy phenotype further, we immunostained endogenous LC3 in HeLaM cells transfected with a non‐targeting siRNA or siRNA to deplete VPS16, VPS33A, VIPAR or VPS33B (Figure 4B,C). Quantification of the fluorescence signal confirmed a significant accumulation of LC3‐positive autophagosomes after depletion of VPS16 or VPS33A when compared to control cells treated with non‐targeting siRNA. No autophagosome accumulation was observed after depletion of VIPAR or VPS33B (Figure 4B,C). In these experiments we observed a greater accumulation of LC3‐positive structures following depletion of VPS16 than after depletion of VPS33A (Figure 4B,C). The results show that VPS33A and VPS16, but not VIPAR and VPS33B, are required for the fusion of autophagosomes with lysosomes.

### Recruitment of VPS33A to the HOPS complex via interaction with VPS16 is required for autophagosome–lysosome fusion

**Figure 5:**
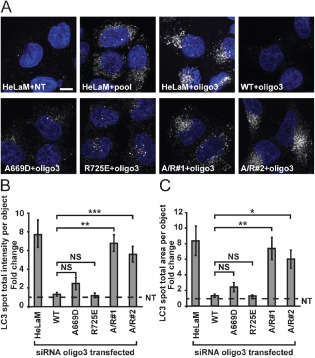
**Association of VPS33A with the HOPS complex is required for autophagosome‐lysosome fusion**. HeLaM cells or HeLaM cells stably expressing HA‐tagged siRNA oligo3‐resistant VPS16 wild type (WT), single mutant (A669D, A725E) or double mutant A669D/R725E (A/R, independent clonal cell lines #1 and #2) were transfected with non‐targeting (NT), VPS16 pool or VPS16 oligo3 siRNA oligonucleotides at 100 nm. A) Representative confocal images of fixed cells that were immunostained for endogenous LC3 (white). DNA (blue) was labelled with Hoechst 33258. Scale bar: 10 µm. B and C) Cells were subjected to automated measurement of LC3 signal intensity using a Cellomix® ArrayScan™ VTi microscope. B) LC3 spot total intensity per object. C) LC3 spot total area per object. For (B) and (C) fold changes were calculated between NT and siRNA oligo3 transfected cells (depletion of endogenous VPS16) of the same genotype. Dotted line indicates levels of NT‐transfected controls. Means ± SD of at least 4500 cells per condition from three independent experiments performed in triplicate are shown. *p < 0.02, **p < 0.01, ***p < 0.008, NS, not significant using two‐tailed unpaired t‐test.

To test whether the interaction of VPS33A with the rest of HOPS via binding to VPS16 is required for autophagosome–lysosome fusion we immunostained endogenous LC3 in HeLaM cells depleted of endogenous VPS16 but stably expressing siRNA‐resistant HA‐VPS16(WT), HA‐VPS16(A669D), HA‐VPS16(R725E) or HA‐VPS16(A669D/R725E). Images obtained by confocal microscopy and quantification of LC3 fluorescence on images captured by automated microscopy showed that expression of HA‐VPS16(WT) rescued the block in autophagosome–lysosome fusion observed upon VPS16 knock‐down in HeLaM cells (Figure 5A–C). Expression of both single mutants, VPS16(A669D) and HA‐VPS16(R725E), showed a tendency towards the cells having more autophagosomes in images taken by confocal microscopy (Figure 5A). However, when we quantified LC3 staining by automated microscopy there were no significant differences in the ability of VPS16(A669D) or HA‐VPS16(R725E) to rescue the block in autophagosome–lysosome fusion when compared with HA‐VPS16(WT) (Figure 5B,C). As discussed above, this is probably due to some residual binding activity of the single mutants to VPS33A that was below the sensitivity of detection by immunoprecipitation. Stable expression of the double mutant HA‐VPS16(A669D/R725E) failed to rescue the block in autophagosome–lysosome fusion observed upon VPS16 knock‐down. Thus, the recruitment of VPS33A to the HOPS complex via its interaction with VPS16 is required for autophagosome–lysosome fusion in HeLaM cells.

### VIPAR and VPS33B are not part of the HOPS complex

**Figure 6:**
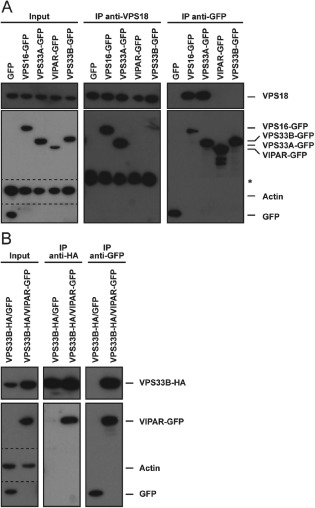
**VIPAR and VPS33B are not part of the HOPS complex**. Immunoprecipitation followed by SDS PAGE and immunoblotting. A) Lysates of HeLaM cells transiently expressing GFP or stably expressing VPS16‐GFP, VPS33A‐GFP, VIPAR‐GFP or VPS33B‐GFP were subjected to immunoprecipitation with anti‐VPS18 antibody (middle) or GFP‐TRAP® agarose beads (right). Dotted line indicates separate images arising from different exposure times of membrane. *Heavy chain of anti‐VPS18 antibody used for immunoprecipitation detected by cross reactivity with HRP conjugated, goat‐anti‐rabbit IgG secondary antibody. B) Lysates of HeLaM cells stably expressing VPS33B‐HA that were transiently transfected with empty GFP vector or stably coexpressing both VPS33B‐HA and VIPAR‐GFP were subjected to immunoprecipitation with anti‐HA affinity matrix (middle) or GFP‐TRAP agarose beads (right).

Our experiments showed that all HOPS proteins, but not VIPAR or VPS33B, are required for the delivery of endocytic cargo to lysosomes and for autophagosome–lysosome fusion (Figure 1). To test whether VIPAR or VPS33B is physically present in the HOPS complex, lysates from HeLaM cells stably expressing C‐terminally GFP‐tagged VPS16, VPS33A, VIPAR, or VPS33B, or transiently expressing GFP alone, were incubated with an antibody against endogenous VPS18 (Figure 6A). Co‐immunoprecipitation of VPS18 was observed with VPS16‐GFP and VPS33A‐GFP, but not with VIPAR‐GFP, VPS33B‐GFP or GFP. The reciprocal experiment, in which the GFP‐tagged proteins were captured on a GFP affinity matrix, confirmed these results as only VPS16‐GFP and VPS33A‐GFP co‐immunoprecipitated endogenous VPS18 but no interaction of VIPAR‐GFP or VPS33B‐GFP with endogenous VPS18 was detected. These results were supported by liquid chromatography tandem mass‐spectrometry analysis after immunoprecipitation of endogenous VPS18 from HeLaM cells. In this experiment, peptides of VPS11, VPS16, VPS18, VPS33A and VPS41 were detected but no peptides of VIPAR or VPS33B could be found (data not shown). Immunoprecipitation experiments from lysates of HeLaM cells stably expressing both VPS33B‐HA and VIPAR‐GFP with either HA or GFP affinity matrices revealed interaction of both proteins (Figure 6B). Taken together our results show that human VIPAR and VPS33B are neither present in HOPS nor CORVET, but form an independent complex.

## Discussion

In this study, we investigated the requirement for HOPS proteins in the final stages of delivering endocytosed cargo from endosomes to lysosomes and the fusion of autophagosomes with lysosomes in mammalian cells. Our data confirm and extend previous studies showing a role for mammalian HOPS proteins in these processes by establishing that all the six HOPS proteins (VPS11, VPS16, VPS18, VPS33A, VPS39 and VPS41) are essential for fusion of endosomes with lysosomes. Whilst the requirement for all the HOPS proteins for fusion with vacuoles or lysosomes is well established in yeast and *Drosophila melanogaster* [reviewed in 29, 36, 50, 51], this has had hitherto not been shown in mammalian cells in a single study. Prior functional studies on the late endocytic pathway in mammalian cells either investigated subsets of, or individual, HOPS proteins using over‐expression or depletion approaches 17, 52, 53. In the case of the mammalian autophagic pathway, whilst previous studies have implicated the requirement for HOPS proteins in autophagosome–lysosomes fusion, there has been contradictory evidence concerning the requirement for VPS16 27, 28, 46. Our data implicate the entire HOPS complex as being essential for the final stages of delivery to mammalian lysosomes.

We exploited our atomic‐resolution structure of the human VPS33A/VPS16 complex 44 to test the effects upon HOPS function of tandem mutations in VPS16 (A669D/R725E) that prevent binding to VPS33A or in VPS33A (K429D/I441K, Y438D/I441K and K429D/Y438D) that prevent binding to VPS16. Using depletion/rescue experiments, we observed that the VPS33A or VPS16 double mutants were unable to support fusion of lysosomes with endosomes and we also found that the VPS16 double mutant did not support fusion of lysosomes with autophagosomes. Taken together, these experiments showed that recruitment of the SM protein VPS33A to the remainder of the HOPS complex is essential for both these fusion events. Unusually amongst SM proteins, VPS33A lacks a binding site for syntaxin SNARE protein N‐terminal peptides 44, 54. The requirement for VPS33A binding by VPS16 for correct HOPS function is consistent with the hypothesis proposed for yeast HOPS that the complex plays the role of the syntaxin N peptide in other SNARE/SM complexes and recruits VPS33A to appropriate sites of SNARE‐mediated membrane fusion within the cell 55. This recruitment of VPS33A, and thus SM protein activity, would allow mammalian HOPS to contribute to catalysis of trans‐SNARE complex assembly, protection of SNARE complexes from disassembly and to play a role in lowering the energy barrier for membrane fusion as suggested for yeast HOPS 10, 11, 12, 55, 56. In our experiments using cells transfected with pLXIN constructs to stably overexpress VPS33A or VPS16 we did not observe any obvious clustering of endocytic organelles by immunofluorescence microscopy, as previously reported when other HOPS proteins were transiently overexpressed 52, 53. This is probably due to the presence of a weaker promoter in pLXIN and consequent lower levels of protein expression than in the previous studies, in which plasmids with strong promoters were used. In our depletion/rescue experiments, the amount of endosome–lysosome fusion was even greater than in control cells when we rescued with wild‐type HA‐VPS16 (Figure 2B). We cannot exclude the possibility that VPS16 is limiting for the assembly of functional HOPS complexes and that the increase in fusion is caused by modest overexpression of wild‐type HA‐VPS16. We were unable to test this hypothesis because no suitable, specific VPS16 antibodies were available.

Our depletion experiments provide compelling evidence that neither VPS33B nor the VPS16 homolog VIPAR are required for fusion of lysosomes with either endosomes or autophagosomes. Moreover, we were unable to co‐immunoprecipitate these proteins with endogenous VPS18 when tagged versions were stably over‐expressed in HeLaM cells, in contrast to the co‐immunoprecipitation of tagged VPS16 and VPS33A with endogenous VPS18. Previously, a very low level of co‐immunoprecipitation of co‐expressed, tagged VIPAR and VPS33A was observed in HeLa cells 41. In HEK cells, co‐immunoprecipitation of co‐expressed tagged VIPAR and other individual HOPS proteins was observed and co‐sedimentation, in the presence of a cross‐linker, of VPS16 and VPS18 with endogenous or tagged VIPAR was also detected 41. Although those results appear to be at odds with our present findings, we stress that our experiments cannot rule out a very low level and/or low affinity interaction of VIPAR and/or VPS33B with HOPS proteins. However, the experiments described above together with those reported previously by ourselves and others 44, 57, 58, as well as the observation that VIPAR and VPS33B cluster together but separately from the HOPS proteins using the proteomic technique of fractionation profiling 59, argue strongly that in mammalian cells VIPAR and VPS33B bind to each other in a stable complex separate from that formed by the HOPS proteins. Given the lack of robust interactions with VPS18 we also consider it unlikely that VPS33B is the SM protein for mammalian CORVET as has been proposed recently for *C. elegans* 38. Our hypothesis that mammalian VIPAR and VPS33B are not part of HOPS or CORVET is supported by previous evidence that these two proteins have different functions to their HOPS paralogs in the biogenesis of LROs 20, 21, 41, 57, 60, 61, 62, 63, and that mutations in the genes encoding VPS33B or VIPAR cause a multisystem disorder named arthrogryposis, renal dysfunction and cholestasis syndrome 58, 64. Even in cells without specialized LROs, the localization of VIPAR/VPS33B may define a specialized endocytic compartment 40, 41, possibly on a recycling pathway to the cell surface, and thus depletion of these proteins could have pleiomorphic effects on endocytic traffic and endosome morphology despite not being required for the final stages of delivery to lysosomes.

In summary, our data strongly support a model in which the whole mammalian HOPS complex is required for endocytic delivery to lysosomes. The binding of the SM protein VPS33A to the remainder of the HOPS complex is essential for the fusion of lysosomes with endosomes or autophagosomes. Our data are consistent with a model derived mostly from studies in yeast in which HOPS is required for tethering events and interactions with SNARE proteins prior to membrane fusion late in the endocytic and autophagic pathways. We found no evidence that the VPS16 and VPS33A paralogs VIPAR and VPS33B were part of the stable HOPS complex in mammalian cells nor that they had any role late in the endocytic or autophagic pathways.

## Materials and Methods

### Mammalian cell culture, microscopy and FACS analysis

HeLaM cells were cultured in RPMI supplemented with 10% (v/v) foetal bovine serum (FBS), 2 mm glutamine, 100 U/mL penicillin and 100 µg/mL streptomycin. HeLaM cells stably expressing HA‐VPS16(WT), HA‐VPS(A669D), HA‐VPS16(R725E) 44, HA‐VPS16(A669D/R725E), VPS33A‐HA(WT), VPS33A‐HA(K429D/I441K), VPS33A‐HA(Y438D/I441K), or VPS33A‐HA(K429D/Y438D), VPS16‐GFP, VPS33A‐GFP, VIPAR‐GFP, VPS33B‐GFP, VPS33B‐HA, or both VPS33B‐HA and VIPAR‐GFP, were generated using the pLXIN retroviral system (Clontech) as previously described 65. HeLaM cells stably expressing mRFP‐GFP‐LC3 were as described in 47.

To assess autophagy by immunocytochemistry, cells were plated on glass coverslips, fixed in 3.4% formaldehyde and permeabilized in 0.1% Triton X‐100 in phosphate buffered saline (PBS), pH 7.4. Fixed cells were blocked with 3% bovine serum albumin (BSA) in PBS before incubation with primary LC3 antibody (MBL) followed by AlexaFluor488‐conjugated secondary antibody (Invitrogen) and Hoechst 33258 staining. Images were acquired on a Zeiss LSM710 confocal microscope with Zeiss zen software. Total LC3 fluorescence intensity from at least 10 fields (≥5 cells per field) for each of three independent experiments was measured using imageJ software package and normalized to cell area. Automated quantification of LC3 spot total intensity and total area per object was performed on cells cultured in black PerkinElmer 96‐well view plates using a Cellomix® ArrayScan™ VTi High Content Screening Microscope (Cellomics) and the Spot Detector V4 algorithm. In short, immunohistochemistry was performed and nuclei were labelled with Hoechst 33258 to identify individual cells. Flow‐Check™ 675 fluorospheres [APC (675/633) Set‐up Kit, Beckman Coulter] were used to adjust the plane of focus and the total fluorescence intensity or total area of LC3 spots per cell was measured from at least 3 wells, with ≥500 cells per well for each condition. Fold changes were calculated by dividing results from siRNA oligo3 transfected cells by the results of non‐targeting siRNA treated controls of the same genotype. Results presented are from three independent experiments and significance was calculated by two‐tailed *t*‐test with pairing of samples from individual experiments.

To measure the delivery of endocytosed dextran to lysosomes by quantitative live cell confocal microscopy, cells were first loaded for 2 h with 1 mg/mL dextran Alexa Fluor 488 10 000 MW, anionic, fixable (Life Technologies) in RPMI followed by a chase for 1 h in dextran‐free RPMI. Cells were then rinsed with PBS, incubated in Magic Red® MR‐(RR)_2_ Cathepsin B substrate (1:2600 dilution) (ImmunoChemistry Technologies) in Live Cell Imaging Solution (Molecular Probes® Life Technologies). Magic Red® is a membrane permeable substrate for the lysosomal acid hydrolase Cathepsin B. The bi‐substituted cresyl violet groups in Magic Red® are non‐fluorescent until they are cleaved at one or both arginine amide linkage sites by Cathepsin B in lysosomes 66. After cleavage mono‐ and non‐substituted cresyl violet emits red fluorescence when exited at 550–590 nm. After adjustment to 37°C in the heating chamber of a Zeiss inverted confocal microscope, single confocal images corresponding to 1 Airy unit were taken (three independent experiments with ≥5 fields each per condition, average of 1–2 cells per field, randomly selected). The degree of colocalisation of two channels reflecting the fraction of dextran Alexa Fluor 488 colocalising with Magic Red® was measured by Manders M_1_/M_2_ colocalisation coefficients 67 using zen software (Carl Zeiss). For analysis of total dextran Alexa Fluor 488 or Magic Red® fluorescence, cells were treated as described above and subjected to FACS analysis by a five‐laser LSR Fortessa (BD Biosciences) and the results of 30,000 cells per condition in each of three independent experiments were analysed with flowjo software.

### siRNA‐mediated knock‐down

siRNA oligonucleotides targeting human gene products were designed and synthesized by Dharmacon/Thermo Scientific. HeLaM cells were transfected (two transfections separated by 48 h) using Oligofectamine (Life Technologies) according to the manufacturer's protocol. On‐Target Plus oligonucleotide catalogue numbers were as follows: non‐targeting (NT) control (D‐001810‐01), VPS11 SMARTpool (L‐007022‐00), VPS16 SMARTpool (L‐013003‐01), VPS18 SMARTpool (L‐013178‐00), VPS33A SMARTpool (L‐013330‐01), VPS33A (J‐013330‐10‐0002; oligo2 target sequence is 5′‐GAAGAAACGUCAACCGGGA), VPS39 SMARTpool (L‐014052‐01), VPS41 SMARTpool (L‐006972‐00), VPS33B SMARTpool (L‐007261‐01), VIPAR SMARTpool (L‐016131‐01). siGENOME oligonucleotide catalogue numbers were: VPS16 SMARTpool (M‐013003‐00), VPS16 (D‐013003‐03; oligo3 target sequence is 5′‐AGAAAUCACCCAUUGGCUA). For knock‐down of VPS16 with SMARTpool oligonucleotides, ON‐Target Plus and siGENOME SMARTpool oligonucleotides were mixed at a 1:1 ratio.

### Quantitative real‐time PCR (qPCR)

Total RNAs were isolated from about 1 million cells using the RNeasy Mini Kit (Qiagen). RNA template (1 µg) was used for cDNA synthesis using the QuantiTect Reverse Transcription Kit (Qiagen), which includes depletion of genomic DNA. cDNAs were used for Quantitative PCR (qPCR) using SYBR Green Jumpstart Taq ReadyMix (Sigma‐Aldrich) or SYBR Select Master Mix (Applied Biosystems) on a QuantStudio 6 Flex Real‐Time PCR System (Applied Biosystems). Primers (Table S1) were designed using the NCBI primer‐blast tool (http://www.ncbi.nlm.nih.gov/tools/primer‐blast/). qPCR results were analysed using the comparative ΔΔCt method 68. Non‐targeting siRNA transfected cells were used as a reference sample. Samples of three biologically independent experiments were analysed and the standard deviations of ΔΔCt values calculated.

### Immunoprecipitation and immunoblot analysis

HeLaM cells used for immunoprecipitation experiments were stably expressing VPS16‐GFP, VPS33A‐GFP, VIPAR‐GFP, VPS33B‐GFP, HA‐VPS16, VPS33B‐HA, or both VPS33B‐HA and VIPAR‐GFP, or were transiently transfected with pEGFP‐N1 vector (Clontech) using PEI transfection reagent as previously described 69. Cells were harvested into lysis buffer containing 50 mm Tris–HCl (pH 7.4), 150 mm NaCl, 0.4 or 1% IGEPAL CA‐630 (Sigma‐Aldrich), and Complete protease inhibitors (Roche) and subjected to immunoprecipitation with polyclonal anti‐VPS18 antibody, GFP‐TRAP® coupled agarose beads (Chromotek), or anti‐HA affinity matrix (Roche) as previously described 44. In short, lysates were adjusted to 6 µg/μL total protein and precleared by incubation for 30 min with protein‐A sepharose. For immunoprecipitation precleared lysates were incubated with equilibrated GFP‐TRAP agarose beads (Chromotek) or anti‐HA affinity matrix (Roche) for 3 h at 4°C. For immunoprecipitation with an anti‐VPS18 antibody, precleared lysates were incubated with anti‐VPS18 antibody for 2 h at 4°C before equilibrated protein‐A sepharose was added for 1 h to enable capture of antibody‐protein complexes. Protein samples were boiled for 10 min before being subjected to SDS‐PAGE and immunoblot analysis on Immobilon‐PVDF membrane (Millipore) using the Mini‐Protean and Mini‐Trans‐Blot systems (BioRad) following the manufacturer's protocol. To quantitate VPS33A levels X‐ray films were scanned and subjected to densitometric analysis using imagej software. Each VPS33A signal was normalized to the Actin signal of the respective sample.

### Antibodies

The antibodies used were: Polyclonal rabbit anti‐VPS18 and polyclonal rabbit anti‐VPS33A [both as described 44], polyclonal rabbit anti‐Actin (A2066; Sigma‐Aldrich), monoclonal mouse anti‐HA (HA.11, MMS‐101R; Covance), polyclonal rabbit anti‐GFP 70, monoclonal mouse anti‐LC3 (4E12, M152‐3, MBL, used for immunofluorescence staining), polyclonal HRP conjugated goat‐anti‐rabbit IgG (A9169; Sigma‐Aldrich) and polyclonal HRP conjugated rabbit‐anti‐mouse IgG (A9044; Sigma‐Aldrich).

## Supporting information


**Table S1. Primers for quantitative real‐time PCR (qPCR)**.

**Figure S1. Components of the human HOPS complex but not VIPAR or VPS33B are required for efficient late endosome‐lysosome fusion**. HeLaM cells were transfected with siRNA oligonucleotides at 100 nm and loaded with 10,000 MW dextran Alexa Fluor® 488 (green) for 2 h. After a chase in medium free of fluorescent dextran for 1 h, lysosomes were stained with Magic Red® (red) and cells subjected to live‐cell confocal imaging. Shown are total images of the representative blow‐ups (right) that were shown in Figure 1C. Scale bar: 10 µm.
