# Supplementary material for: Recruitment of VPS33A to HOPS by VPS16 Is Required for Lysosome Fusion with Endosomes and Autophagosomes
Source: Traffic. 2015 Apr 30;16(7):727–42. doi: 10.1111/tra.12283 (PMC4510706; doi:10.1111/tra.12283)
Supplement: Supplementary file 1 — Table S1. Primers for quantitative real‐time PCR (qPCR). [file tra0016-0727-sd1.doc]

**Table S1. Primers for Quantitative Real-Time PCR (qPCR)**

| Gene | Sequence 5’-3’ |
| --- | --- |
| Actin | Forward ATAGCAACGTACATGGCTGG  Reverse CACCTTCTACAATGAGCTGC |
| VPS11 | Forward AAGAACCTCATGCCACCTCTTC  Reverse GGTAGTCCCTGATGACGGAGA |
| VPS16 | Forward CTTCTTGTTGGCGATGTGGC  Reverse CTCAGCCTCATTCCGGTGTT |
| VPS39 | Forward TCAAGTGCTCAAGAACCTTCTCC  Reverse CCTGCTGGTGTAAAATCCGCT |
| VPS41 | Forward GAAACTGGGTCCCTTGAAGAATCTA  Reverse GCTTGGGTTCCTCTTCGCTC |
| VIPAR | Forward ACCACAAAGAACTGGCTGGG  Reverse GAAGCCAATGGGTGCTCTCT |
| VPS33B | Forward TGCCGAATCATTGAGCAGGT  Reverse GTACCACCTCATCAAGGCCC |
