# Supplementary figures and images for: Recruitment of VPS33A to HOPS by VPS16 Is Required for Lysosome Fusion with Endosomes and Autophagosomes
Source: Traffic. 2015 Apr 30;16(7):727–42. doi: 10.1111/tra.12283 (PMC4510706; doi:10.1111/tra.12283)

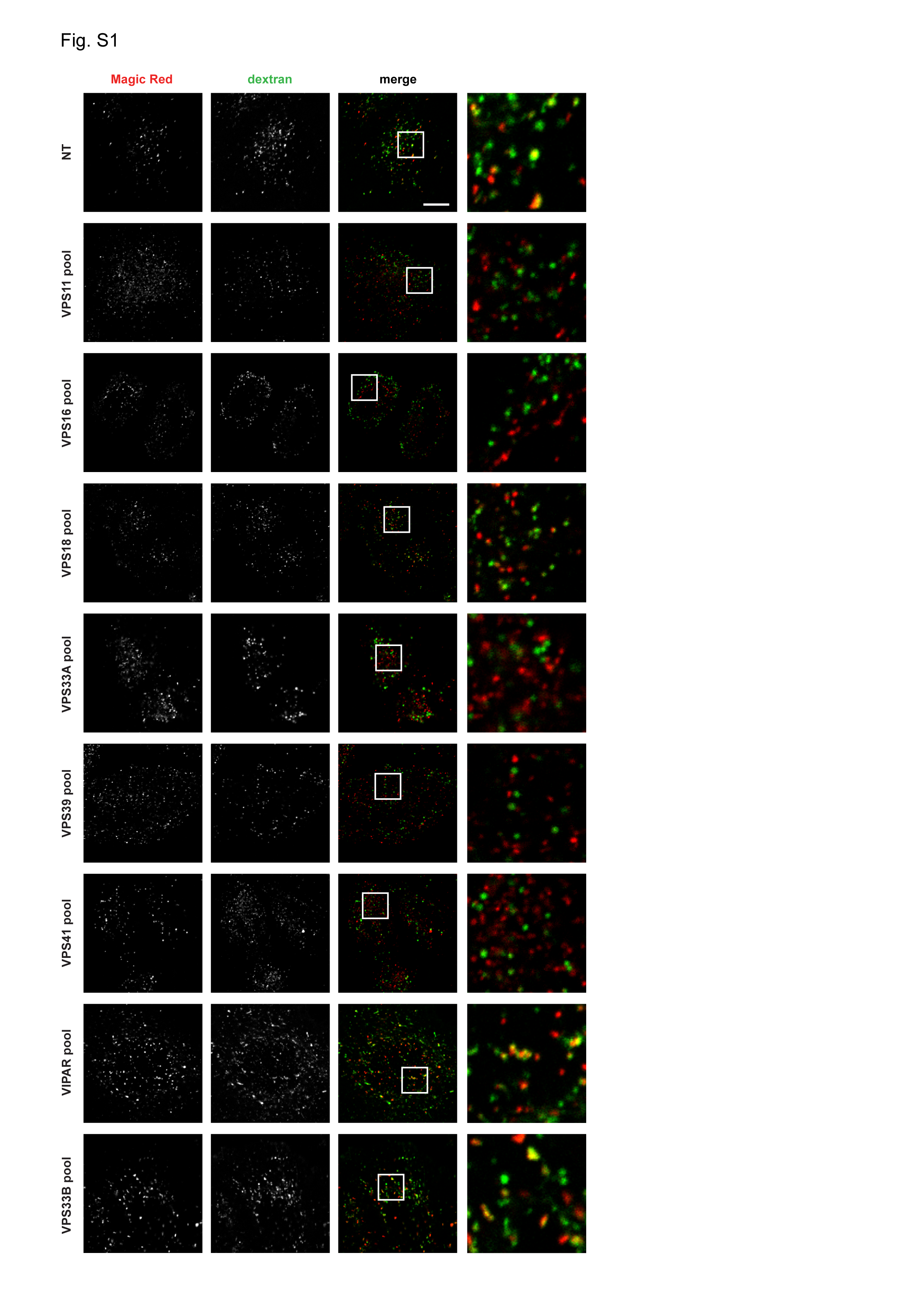

Supplement: Supplementary file 2 — Figure S1. Components of the human HOPS complex but not VIPAR or VPS33B are required for efficient late endosome‐lysosome fusion. HeLaM cells were transfected with siRNA oligonucleotides at 100 nm and loaded with 10,000 MW dextran Alexa Fluor® 488 (green) for 2 h. After a chase in medium free of fluorescent dextran for 1 h, lysosomes were stained with Magic Red® (red) and cells subjected to live‐cell confocal imaging. Shown are total images of the representative blow‐ups (right) that were shown in Figure 1C. Scale bar: 10 µm. [file tra0016-0727-sd2.tif]
